# The regional impact of the COVID-19 lockdown on the air quality in Ji'nan, China

**DOI:** 10.1038/s41598-022-16105-6

**Published:** 2022-07-15

**Authors:** Kun Li, Ruiqiang Ni, Tenglong Jiang, Yaozhen Tian, Xinwen Zhang, Chuanrong Li, Chunying Xie

**Affiliations:** 1 Forestry College of Shandong Agricultural University, Tai’an, 271018 Shandong People’s Republic of China; 2 Mountain Tai Forest Ecosystem Research Station of State Forestry Administration/Key Laboratory of State Forestry Administration for Silviculture of the Lower Yellow River, Tai’an, 271018 Shandong China; 3 Jinan Eco-environmental Monitoring Center of Shandong Province, Ji’nan, 250014 Shandong China

**Keywords:** Ecology, Environmental sciences

## Abstract

A number of strict lockdown measures were implemented in the areas most affected by COVID-19 in China, including Ji’nan city, from 24 January to 7 February 2020. Due to these forced restrictions, the pollution levels in cities across the country drastically decreased within just a few days. Since traffic pollution and industrial emissions are important factors affecting regional air quality, congestion has a significant impact on the environment. Therefore, using the aid of air quality data for six pollutants (PM_10_, PM_2.5_, SO_2_, NO_2_, CO and O_3_) from 11 monitoring stations (located in urban, suburban and urban-industrial regions) across Ji’nan, we employed the air quality index (AQI) to investigate the spatial pattern of air quality in the pre-COVID-19 (pre-COVID) and COVID-19-related lockdown (COVID lockdown) periods. The results showed that air quality significantly improved during the COVID lockdown period. Among the selected pollutants, compared to the corresponding pre-COVID levels, the greatest reduction was observed for the concentration of NO_2_ (54.02%), while the smallest reduction was observed for the concentration of SO_2_ (27.92%). The PM_2.5_ (38.73%), PM_10_ (44.92%) and CO (30.60%) levels also decreased during the COVID lockdown period; only the O_3_ concentration increased (37.42%) during this period. Overall, air quality improved by approximate improvements of 37.33% during the COVID lockdown period. Approximately 35.48%, 37.01% and 43.43% in the AQI were observed in urban, suburban and urban-industrial regions, respectively. Therefore, the AQI exhibited remarkable regional differences in Ji'nan. This study demonstrates the contributions of the transportation sector and local emissions to improving air quality in typical urban areas, and these research results can provide guidance for the further monitoring of air pollution in northern Chinese cities.

## Introduction

In the past 10 years, air pollution has become a major environmental problem in northern China. From 2013 to 2017, the Chinese government implemented the Air Pollution Prevention and Control Action Plan (APPCAP) to improve the quality of the ecological environment through the adoption of a number of control measures, and air quality has steadily improved in recent years^1^. For example, in 2014–2018, decreasing levels of carbon monoxide (CO), nitrogen dioxide (NO_2_), sulfur dioxide (SO_2_), and inhalable and fine inhalable particulate matter (PM_10_ and PM_2.5_, respectively) were observed at 87%, 63%, 93%, 78% and 89%, respectively, of air quality monitoring stations^2^. Specifically, these measures had a significant impact on reducing PM_2.5_ concentrations. However, many people in China are still exposed to polluted air^3^, which kills nearly 1 million people per year in China^4^. The APPCAP is unlikely to result in further major reductions in annual deaths attributable to PM_2.5_ pollution, and thus, more ambitious measures are needed to reduce the health effects of air pollution by 2030.

In late December 2019, pneumonia caused by a new type of coronavirus was first discovered in Wuhan, Hubei Province, China. The pathogen has been identified as Coronavirus 2 Severe Acute Respiratory Syndrome (SARS-CoV-2), which the World Health Organization (WHO) officially called Coronavirus Disease 2019 (COVID-19)^5^. To date, many studies have confirmed that COVID-19 can be transmitted through the air^6–9^. Thus, control measures could be employed to prevent further COVID-19 infection^10^. To curb the rapid spread of COVID-19, the Chinese government initiated the highest level of emergency response in the national public health emergency response system and implemented a series of strict self-isolation, social distancing, traffic restriction, roadblock and prevention and control measures^11–14^. These controls also limited pollutant emissions and improved air quality to a certain extent, for instance, by reducing the concentration of nitrous oxides (NOx) by 77.3% and that of particulate matter (PM) by 200%^15^. However, previous studies have confirmed that even a substantial reduction in traffic emissions coincident with only a small reduction in industrial emissions will not help prevent serious air pollution in China^16^. For example, the opposite result was confirmed in Hubei, where pollution incidents often occurred due to the regional transmission of upstream air pollutants during the strictest lockdown period^17^.

Air pollutants comprise a mixture of chemical substances, including carbon oxides (COx), NOx, ozone (O_3_), sulfur oxides (SOx) and (PM), which can increase respiratory infections^5,18^. In addition, these ambient air pollutants help promote the spread of COVID-19^5,19,20^, mainly by overexpression of angiotensin‑converting enzyme 2 in respiratory cells^21^. Specifically, northern, one of the most densely populated areas in the world, has experienced heavy fog for the past 20 years^22^. Consequently, it was predicted that COVID-19 would spread at an accelerated rate in this environment. To prevent the rapid spread of COVID-19, strict restrictions (lockdowns), such as factory restrictions and traffic restrictions, have been implemented in various regions^23^. These measures helped reduce of most pollutant emissions, indicating that lockdowns can play a key role as a potential solution for reducing future air pollution^24^. During these lockdowns, once the interfering factors were removed, a significant relationship between air pollution and COVID-19 infection was observed^20^; notably, significant declines in NOx, PM, SOx, and CO and a significant increase in O_3_ were observed during COVID-19-related lockdowns^5,13,25,26^. However, there were obvious differences among different geographical environments. Many studies have shown that restrictions result in both direct and indirect contributions to air quality at the regional and global levels^27^, such as in urban, suburban, and rural areas and in mining regions^24,28^. COVID-19 has clearly had a multiscale effect on air quality. Nevertheless, thus far, it remains unclear how COVID-19 affects air quality at the urban scales and whether different regions have similar pollution characteristics. Most studies have focused on the characteristics of pollutant concentration changes in global or national regions with large spatial scales, but different regional economic structures have led to different decreases in air pollutant emissions and different magnitudes of air pollutant concentration changes during the epidemic control period, while relatively little attention has been paid to the characteristics of spatial pollutant concentration changes in cities where spatial scales require greater refinement.

Ji'nan city, is located in the transition zone between the mountainous area and the northern plain, and the topography is generally high in the south and low in the north, with a shallow basin shape. As the capital of Shandong Province, the economic development in Ji'nan ranks second in Shandong province, accounting for 13.3% of the Province’s GDP in 2019^29^. At the same time, Ji'nan is also one of the 15 subprovincial cities in China, serving as an important economic and transportation hub connecting the northern business district, the southern Yangtze River Delta business district, and the eastern and central parts of the Shandong Peninsula^30^.

Recently, the air quality in Ji'nan has received considerable attention^31^. However, the air pollution problem has long been a vital factor limiting the development of Ji'nan city and surrounding regions. The special topography of Ji'nan city is not conducive to the diffusion of pollutants. The COVID-19 pandemic offers a unique opportunity to assess exactly how reducing local emissions can affect the air quality in megacities^32^. Our study aimed to analyse and assess the characteristics and shifts of air quality in different regions of Ji'nan city during the epidemic control period.

To determine the main changes in the concentrations of PM_10_, PM_2.5_, NO_2_, SO_2_, CO and O_3_ and in the air quality index (AQI), in this study, we systematically analysed continuous 24 h data for particulate and gaseous pollutants at seven urban sites, three urban-industrial sites and one suburban site in Ji'nan. The average of each of these parameters was calculated from January 15–23, 2020, and in the lockout period from January 24 to February 7, 2020. The objective was to evaluate the relative change (%) and the average concentration difference (μg/m^3^) both in each pollutant and in the AQI between the two time periods (before the lockdown and during the lockdown) and among the three regions (urban, suburban and urban-industrial areas). Our results will provide a reference for assessing and formulating scientific emission reduction policies to improve the air quality of Ji'nan City and surrounding regions in the future.

## Results and discussion

### Overall characteristics of air pollutants

**Figure 1:**
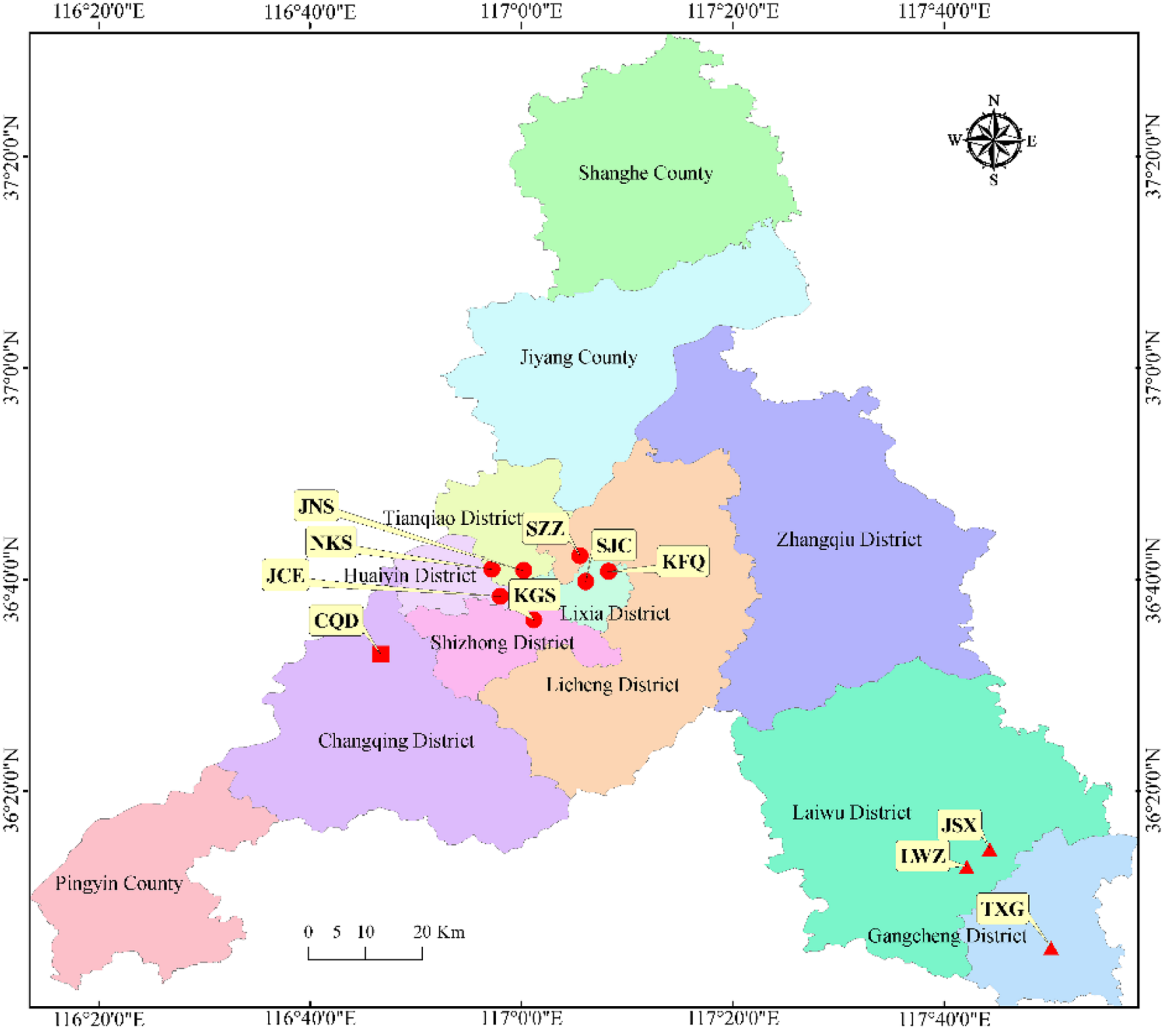
Spatial distributions of the different observation sites and industrial enterprises above a designated size threshold in Ji'nan city. JCE, machine tool factory No. 2; LSX, technical college; JNS, Ji’nan fourth building group; KFQ, economic development zone; KGS, Kegansuo; LWZ, Laiwu memorial hall; NKS, Agricultural Scientific Institute; SZZ, Seed warehouse of Shandong Province; SJC, Ji’nan monitoring station; TXG, Taixing company; CQD, Changqing school. Red circles, red triangles and red squares represent stations in urban, urban-industrial and suburban regions, respectively. The map of Observation site was completed by the geostatistical analysis module of ArcGIS (version 10.3, https://developers.arcgis.com/).

The results of previous studies indicated that local pollution is highly important in determining the emissions of air pollutant. Therefore, in this study, we estimated the changes in pollution and the AQI between the pre-COVID and COVID lockdown periods and among the different regions in Ji'nan. A comparison of the different pollutant concentrations analysed in this study shows that the concentrations of almost all pollutants decreased during the COVID lockdown period; only the concentration of O_3_ increased continuously as the COVID lockdown period progressed (Fig. 1).

**Figure 2:**
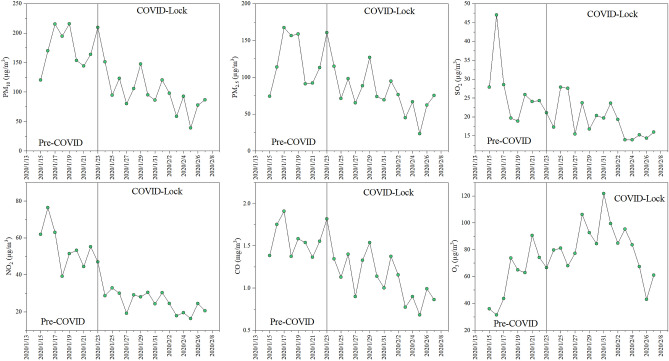
Temporal variations in the mass concentrations of air pollutants (PM_10_, PM_2.5_, NO_2_, SO_2_, CO and O_3_) at the urban site in Ji'nan during the observation period.

During the observation period, the daily average mass concentrations of PM_10_, PM_2.5_, SO_2_, NO_2_, CO, and O_3_ in Ji'nan were 137.09 µg/m^3^, 101.35 µg/m^3^, 22.70 µg/m^3^, 39.77 µg/m^3^, 1.28 mg/m^3^, and 71.84 µg/m^3^, respectively (Fig. 2). The mass concentrations of PM_10_ and PM_2.5_ exceeded the daily average Grade I values (50 µg/m^3^ and 35 µg/m^3^) of the Ambient Air Quality Standard of China (CAAQS, GB 3095-2012) during the whole observation period. In contrast, the mass concentrations of NO_2_, SO_2_, CO and O_3_ were substantially lower than the daily average Grade I values (80 µg/m^3^, 50 µg/m^3^, 4 mg/m^3^ and 100 µg/m^3^, respectively) of the CAAQS each day. During the pre-COVID period, the daily average mass concentrations of PM_10_, PM_2.5_, SO_2_, NO_2_, CO, and O_3_ in Ji'nan were 177.03 µg/m^3^, 125.94 µg/m^3^, 26.39 µg/m^3^, 54.52 µg/m^3^, 1.59 mg/m^3^, and 60.72 µg/m^3^, respectively. The mass concentrations of all these pollutants, except NO_2_, CO and O_3_, exceeded the daily average Grade I values of the CAAQS. The mass concentration trends during the COVID lockdown period were consistent with those during the pre-COVID period, but there were significant differences in the concentrations between the periods. In summary, the air quality in Ji'nan was generally good from January 24 to February 7, 2020, mainly due to the strict prevention and control measures for COVID-19.

### Effects of regional differences and lockdown on air pollutants

**Figure 3:**
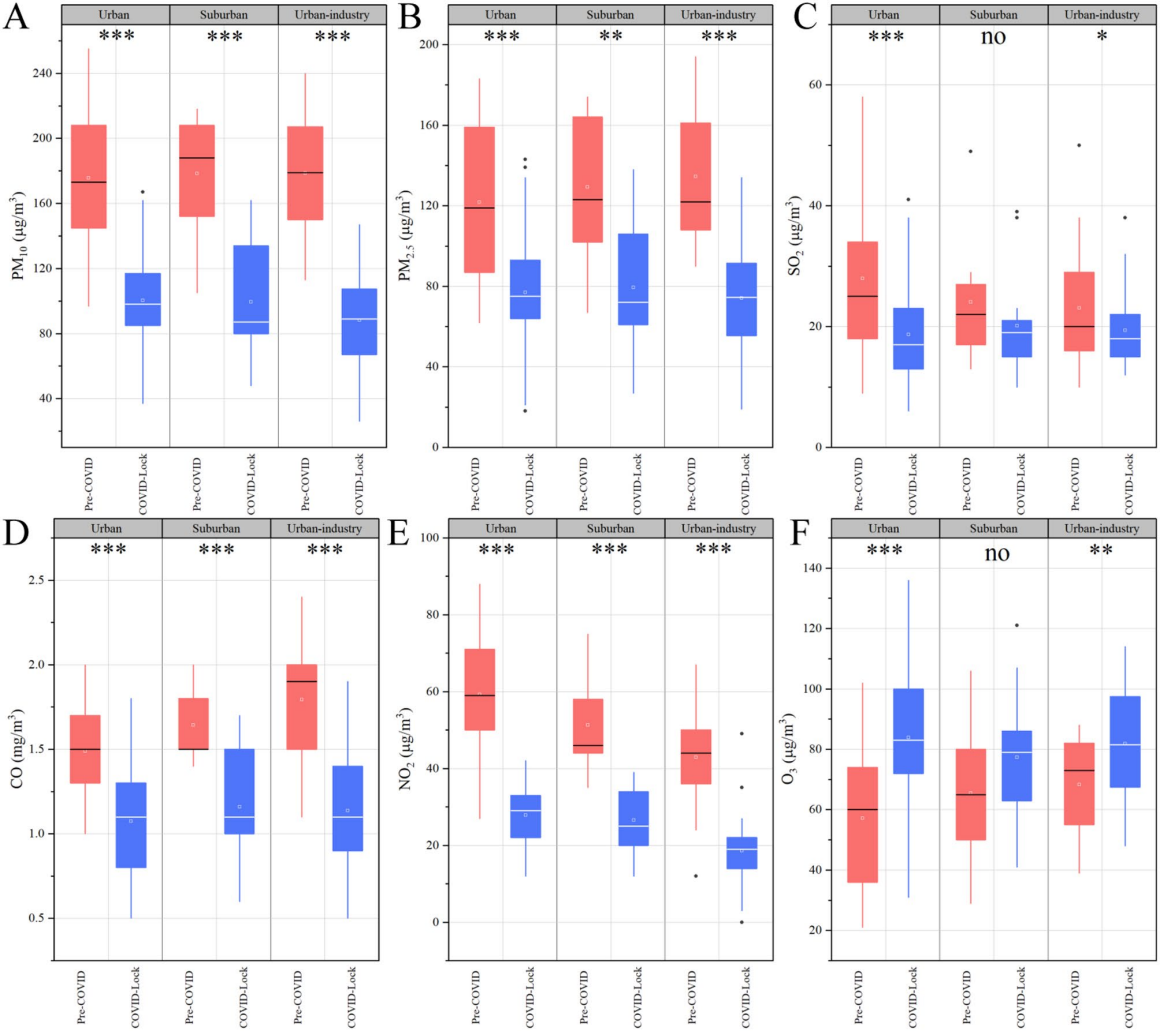
Mean concentrations (± SD, mg/m^3^) of PM_10_, PM_2.5_, NO_2_, SO_2_, CO and O_3_ during the pre-COVID and COVID lockdown periods in 2020; the values were determined by combining the urban, suburban and urban-industrial areas at the regional scale. *, ** and *** represent significant differences between the pre-COVID and COVID lockdown periods in the same region (Duncan test, *p = 0.05; **p = 0.01; ***p = 0.001), with nonsignificant results being excluded.

**Figure 4:**
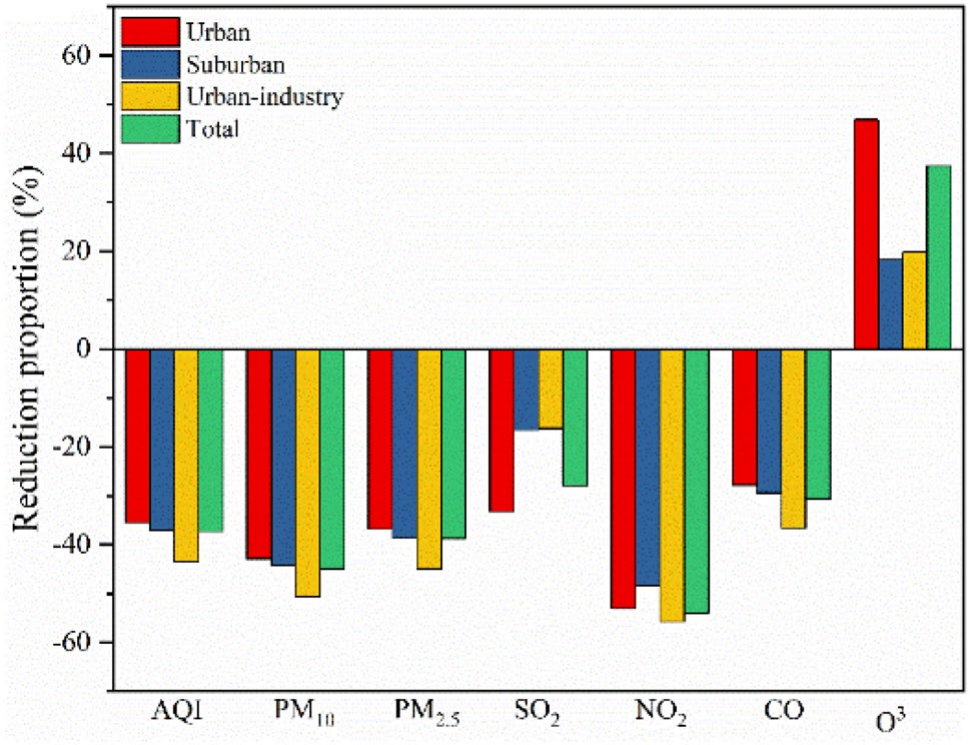
General reductions in the concentrations of major air pollutants.

Our results reveal that the PM_10_, PM_2.5_, NO_2_, SO_2_, CO and O_3_ concentrations in the urban, suburban and urban-industrial regions differed significantly between the COVID lockdown and pre-COVID periods (Figs. 3, 4).

NOx, one of the most important pollutants and a major health hazard, was studied in different countries across the world during COVID-19-related lockdowns. In all three regions studied herein, the highest rate of reduction in NO_2_ concentrations was observed during the COVID lockdown period (Fig. 4), with the NO_2_ levels in the COVID lockdown period being 54.02% on average lower than those during the pre-COVID period (53.07% in urban area, 48.31% in the suburban areas and 55.74% in the urban-industrial area) (Fig. 4); this reduction is greater than that reported at other sites by 26–42%^11^ and 14–38%^18^ but lower than that (50–62%) in Barcelona and Madrid in Spain^33^. As shown in Fig. 3E, the NO_2_ concentrations in the urban, suburban and urban-industrial areas were significantly higher in the pre-COVID period than in the COVID lockdown period, with the pre-COVID the NO_2_ levels in the urban area being 13.46% and 27.63% higher than those in the suburban and urban-industrial areas, respectively. During the COVID-19 lockdown period, the NO_2_ levels in urban areas were 4.69% and 31.75% higher than those in the suburban and urban-industrial areas, respectively. Blocking and controlling the air pollution associated with COVID-19 has helped reduce ground NO_2_ levels^34^ and this effect might be correlated with the tropospheric NO_2_ column density^27^. Among all sources of NO_2_, automobile emissions and power generation are the most important^5^. A systematic review confirmed that a short-term increase in the NO_2_ concentration in urban areas correlates to an increase in the number of pneumonia hospitalizations^5,35^.

The trends in the CO concentration were similar to those in the NO_2_ level. During the COVID-lockdown period, the average CO mass concentrations in the urban, suburban and urban industrial areas were 1.08 mg/m^3^, 1.16 mg/m^3^ and 1.14 mg/m^3^, respectively, which decreased by 27.78%, 29.46% and 36.61%, respectively, compared with those during the pre-COVID period. The highest levels of PM_10_ were also observed during the pre-COVID period in the urban, suburban, and urban-industrial areas in Ji'nan (Fig. 4). The reductions in PM_2.5_ and CO emissions in urban and urban-industrial areas are generally higher than those in suburban areas^25^, supporting our findings. Notably, PM_2.5_ and CO are generated mainly by construction activities and from road dust, natural soil dust and dust from urban-industrial activities^36^. In contrast, the differences in the PM_10_ concentrations among the three regions were not significant during either the pre-COVID period or the COVID-lockdown period (Fig. 3A), which suggests that particles in Ji'nan are strongly diffused. However, the COVID lockdown period had a significant effect on the PM_10_ concentrations, with 42.86%, 44.26% and 50.60% differences in the PM_10_ concentration between the pre-COVID and COVID lockdown periods in the urban, suburban and urban-industrial areas, respectively (average of 44.92%, Fig. 4). The main reasons for the decreases in the concentration of PM were the severe restrictions on vehicle traffic, the cessation of industrial activities, and the stopping of construction projects, which are important sources of floating dust in the urban air^37^. Despite the overall consistency among the observed changes in all regions for the different air pollutants (except O_3_), at the regional level, some differences were statistically significant, while others were not due to the variability among stations, with the differences being more pronounced at the urban, suburban and urban-industrial stations.

O_3_ is a secondary pollutant involved in different atmospheric reaction mechanisms and acts as both a source and sink. Generally, the impact of lockdowns on O_3_ was mixed, with its levels generally falling within ± 20%^38^, but total O_3_ levels remained relatively stable^18^. In this study, by comparing the regional mean concentrations throughout the COVID-19 period, we found that O_3_ concentrations were higher during the COVID lockdown period than during the pre-COVID period, especially in the urban regions (Fig. 3). Furthermore, the mean O_3_ concentration at all stations during the COVID lockdown period was 37.42% higher than that during the pre-COVID period (46.84% in the urban areas, 18.27% in the suburban area, and 19.84% in the urban-industrial areas) (Fig. 4); this finding is consistent with the outcomes of other studies, which reported that O_3_ concentrations increased by (on average) 20% during lockdowns^39^, potentially due, in part, to atmospheric reactivity^37^. The higher lockdown O_3_ concentrations can be attributed to the following three reasons: (1) low PM concentrations can result in more sunlight passing through the atmosphere, encouraging increased photochemical activities and thus higher O_3_ production^40^; (2) a reduction in NOx emissions increases O_3_ formation^41^; and (3) lower PM_2.5_ concentrations means their role as a sink for hydroperoxy radicals (HO_2_) is less effective, which would increase peroxy radical-mediated O_3_ production^42^. During the pre-COVID period, the O_3_ levels were not significantly different among the region, and the same results were observed during the COVID lockdown period. However, in the urban and urban-industrial areas, the O_3_ levels during the COVID lockdown period were significantly higher than those in the pre-COVID period (p < 0.001 and p < 0.01, respectively) (Fig. 3F). Such increases in O_3_ are in line with a 17.56% O_3_ increase in industrial locations in Delhi, India, with minor O_3_ increases of up to 0.78% during the lockdown period^43^.

### Relationships between different pollutants and region in different research stages

**Figure 5:**
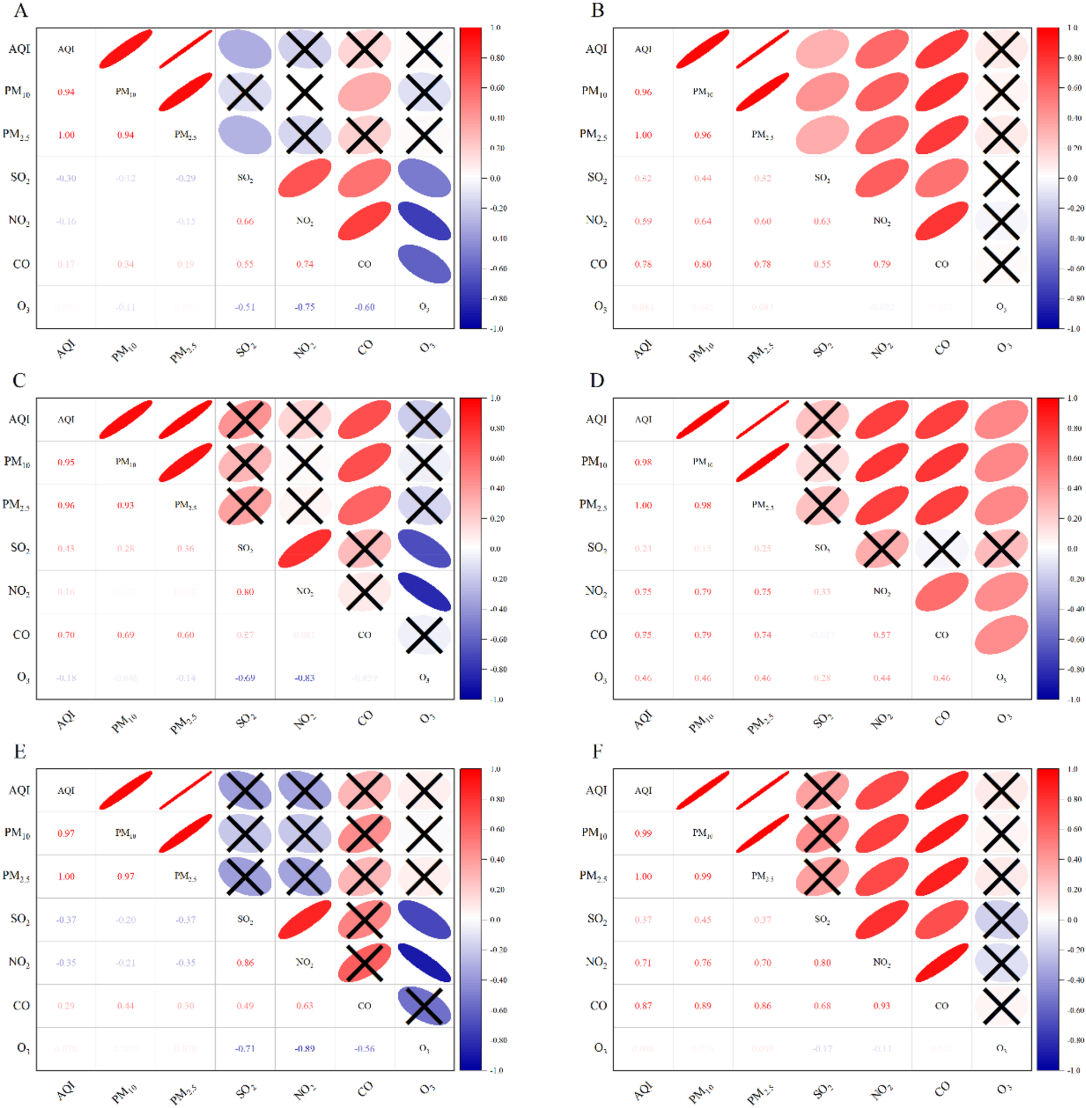
Pearson correlation between various pollutants during different phases and at the different regional sites during the study period. (**A**,**C**,**E**) represent the urban, urban-industrial and suburban areas in the pre-COVID period, respectively, while (**B**,**D**,**F**) represent the urban, urban-industrial and suburban areas in the COVID lockdown period, respectively. × represents a nonsignificant correlation at the 0.05 level.

As particles scatter and absorb sunlight, air pollution reduces the amount of sunlight reaching the Earth. It has been confirmed that reducing PM emissions had the greatest influence on the improvement of air quality during COVID-related lockdowns, but secondary formation was enhanced after these lockdowns ended^13^. These changes affect the distributions of other pollutants. Specifically, PM_2.5_ is best explained by emissions from the transportation and industrial sectors^44^. Some studies have confirmed that PM_2.5_ could trigger COVID-19 spread and increase lethality^45,46^. Therefore, we estimated the relationships of PM_2.5_ with the AQI and the PM_10_, NO_2_, SO_2_, CO and O_3_ concentrations in the air of Ji'nan during the pre-COVID and COVID lockdown periods (Fig. 5). We found that the lockdown affected the relationship between PM_2.5_ and NO_2_, which were relatively weakly or not correlated (r = − 0.15, urban; r = 0.052, urban–industrial; r = − 0.35, suburban) in the pre-COVID period but significantly correlated (r = 0.60, urban; r = 0.75, urban–industrial; r = 0.70, suburban) during the COVID lockdown period. This finding is consistent with the results of Mahato et al.^43^. This clearly means that greater control of regional transport activity is a key factor in reducing pollutant levels as regional transport is completely restricted during the lockdown^43,47^, and there were restrictions on human activities, transportation, and factories during the lockdown period. The increase in the PM_2.5_ concentration may also be due to the incineration of straw and the use of coal-fired power plants in the neighbouring upwind states, leading to the transport of pollutants to urban areas^37^, and may be linked to the distance from coast^48^. Moreover, air temperature is an important parameter that affects the dispersion of air pollutants. An increase in air temperature due to a rise in solar radiation also allows more sunlight to pass through the atmosphere, encouraging increased photochemical activities and thus enhancing O_3_ production^40^. In this study, we found that the average temperatures during the pre-COVID period and COVID lockdown were 2.75 °C and 2.91 °C, respectively. This also indirectly confirms the influence of meteorological conditions on air quality during the lockdown period.

### COVID-19-related lockdowns can improve the air quality index

**Figure 6:**
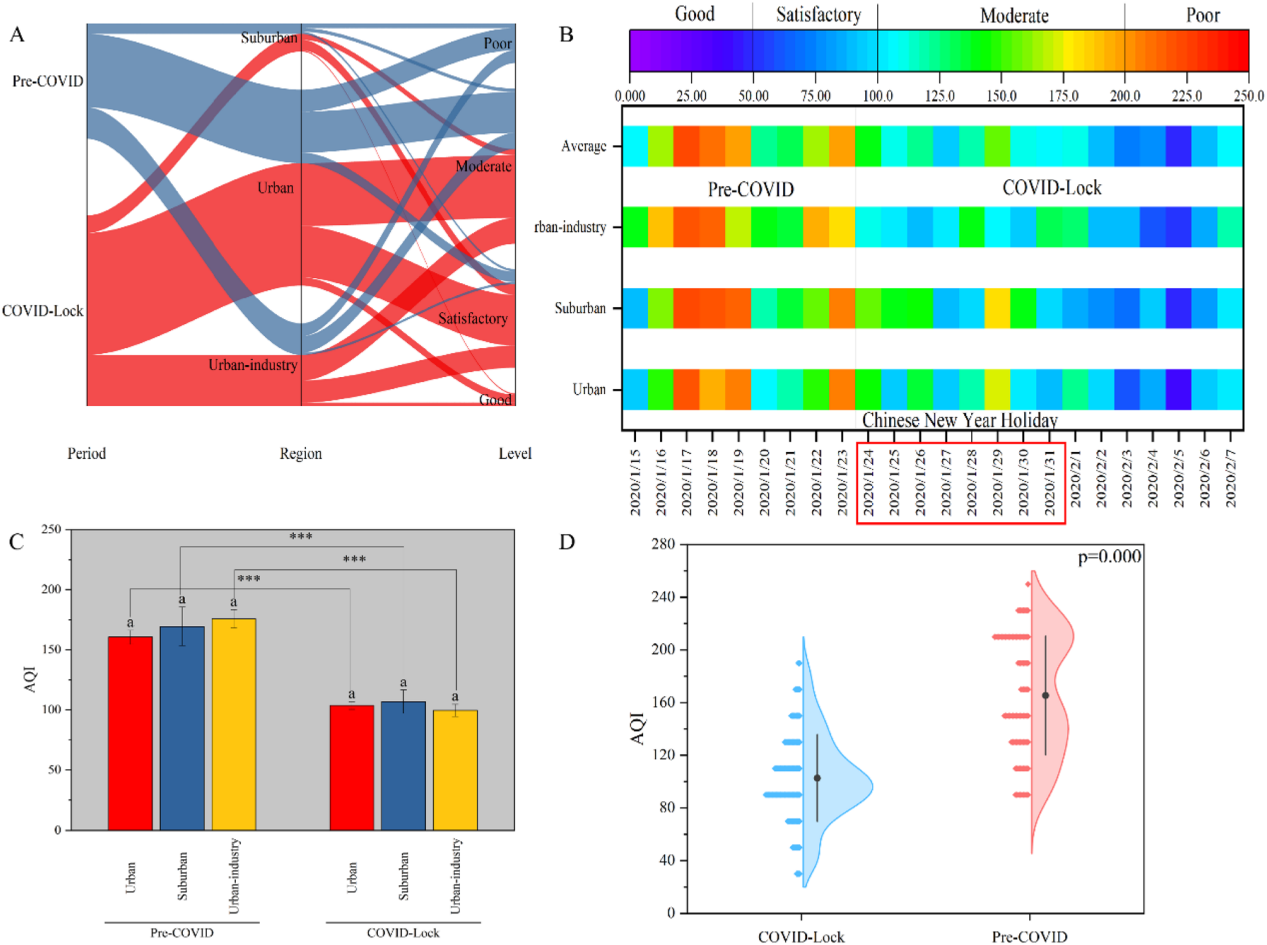
AQI values determined from data collected at the Ji'nan monitoring stations from 15 January to 7 February. (**A**) The parallel plot shows how the AQI level differs among the urban, suburban, urban-industrial areas. (**B**) AQI values determined as time series; (**C**) comparison of the AQI levels among different regions during the pre-COVID and lockdown period; (**D**) comparison of AQI between the pre-COVID and lockdown periods.

**Figure 7:**
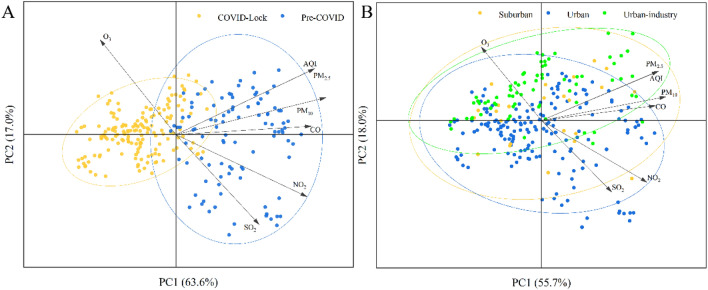
Principal component analysis (PCA) of air pollutants during the pre-COVID and COVID lockdown periods (**A**) and in urban, suburban and urban-industrial areas (**B**).

Figure 6 shows the change in the AQI and the corresponding dominant distribution characteristics during the analysis period. Nations have implemented various containment measures during the COVID-19 pandemic that have resulted in both positive and negative environmental impacts^49^. Nevertheless, a significant improvement in the AQI was observed in the COVID lockdown period in this study. This improvement was caused by a reduction in emissions from the transportation and industry sectors^44,50^. During the pre-COVID period, the AQI was generally poor and moderate in all regions, while during the COVID lockdown period, the AQI was generally good, ranging from satisfactory to moderate (Figs. 6A, 7B). Although there was still considerable room for improvement during the COVID lockdown period, the urban-industrial regions had the best air quality in Ji'nan; indeed, only urban-industrial regions obtained an AQI lower than 100 (Fig. 6C). In areas affected by the transportation and industrial sectors, air quality improved by nearly 60%^43^, especially up to 75% in Tehran^51^. In this study, a 37.33% reduction in the AQI was observed during the COVID lockdown period compared to the pre-lockdown period (Fig. 6D). Approximate reductions of 35.48%, 37.01% and 43.43% were observed in the urban, suburban and urban-industrial regions, respectively, and there was a significant difference in the AQI between the COVID lockdown and pre-COVID periods (Fig. 6C). Thus, the high AQIs at the different sites during the implementation of government intervention measures may have been influenced primarily by heavy pollution from industrial sources^52^. Therefore, the AQI showed remarkable regional differences due to industrial emissions in Ji'nan, where the COVID-19-related lockdown prevented these emissions.

### Principal component analysis (PCA)

To identify the effects of the different regions and lockdown measures on the air pollutant concentrations, an ordination diagram of air pollutants based on PCA was created using Canoco 5.0, as shown in Fig. 7. In this figure, features with narrow included angles are positively correlated, features at wide angles are negatively correlated, and features at a right angle to one another are not correlated; additionally, the neighbourhood distance between factors is similarly used to calculate the corresponding area and congestion period. Figure 7A illustrates two main patterns of the influence of the COVID lockdown on air pollutants. The first two axes explained approximately 80.6% of the total variance (63.6% by PC1 and 17.0% by PC2) in the pollutant concentrations during the COVID lockdown period (Fig. 7A). The lockdown of the city and the shutdown of industrial regions prevented the spread and emission of pollution. At the regional scale (Fig. 7B), the first two axes explain 73.7% of the total variance (55.7% by PC1 and 18.0% by PC2) in the COVID lockdown period; these results indicated that regions changed the distance pattern and accelerated the movement of pollutant patterns. In urban regions, SO_2_ and NO_2_ were the main pollutants, while in suburban and urban-industrial regions, PM_2.5_ was the main pollutant. Previous studies have confirmed that the primary anthropogenic sources of PM, CO, NO_2_, and SO_2_ are the burning of fossil fuels in vehicles and the regional transport of these pollutants due to stubble burning in upwind areas; a combination of both of these sources can also occur^37,53^. In this study, the air pollutants exhibited clear patterns during the pre-COVID and COVID lockdown periods in Fig. 7B. The monitoring stations can be clearly separated during the pre-COVID and COVID lockdown periods, but during the COVID lockdown period, stations cannot be distinctly separated at the regional scale. PM_2.5_ and SO_2_ were the main urban-industrial and urban pollutants, respectively. Overall, the concentrations of PM_2.5_, PM10, CO, NO_2_, SO_2_ and AQI were positively related to the COVID lockdown period, whereas the O_3_ concentration showed a negative association with the COVID lockdown period. Numerous studies suggest that the lockdown measures related to COVID-19 pandemic caused significant decreases in the concentrations of PM_2.5_, NO_2_, PM_10_, SO_2_ and CO globally while O_3_ concentration increased^54,55^. In our study, the results are quite in accord with these conclusions.

## Conclusion and suggestions for intervention

During the COVID lockdown period, human activities were greatly reduced, causing significant reductions in industrial operations and traffic activities, which further led to emissions reductions. In this study, we used the city of Ji'nan as a case study to investigate the effects of the COVID lockdown on air quality. We found that different air pollutants reacted significantly differently to the restrictions.

1. The air quality in Ji'nan improved considerably during the COVID lockdown, with the NO_2_ exhibiting the greatest reduction among all six standard pollutants (excepting O_3_).
2. The Pearson correlation differed in different periods; i.e., PM_2.5_ was not correlated with NO_2_ during the pre-COVID period but was significantly correlated with NO_2_ during the COVID lockdown period.
3. The regional blocking of air pollution can have a substantial impact on urban industrial areas.
4. The implementation of lockdown measures had a more significant effect on the pollutant concentrations than the region.

### Suggestions for intervention

Our analysis has several limitations. Due to a lack of data, we were unable to study the impacts of all the elements of the crisis in the context of the city-scale response. Therefore, it is not clear which elements of the national emergency measures were most effective in reducing air pollution.

Moreover, lockdowns limit emissions and the spread of air pollutants over only a short period; in the long run, we should focus on how to build an ecological city. Pollution management systems should be implemented at different scales, blocking off the sources and transmission paths of different pollutants. Additionally, the Chinese government is carrying out projects to protect and restore the ecologies of full-array ecosystems (mountains, rivers, forests, farmlands, lakes, and grasslands) and systematically resolving ecological environmental problems. The ecological protection and restoration of entire ecosystems are important for constructing an ecological civilization and constitute effective measures for promoting green development; thus, corresponding steps are needed to promote ecological, environmental and economic development.

## Materials and methods

### Observation sites

Ji'nan is located in northern China and includes eleven observation sites covering the urban area (JNS, NKS, JCE, KGS, SZZ, SJC and KFQ), urban-industrial area (JSX, LWZ and TXG), and suburban area (CQD) (Fig. 1). Urban land is mainly located in the city centre, which is the area with the highest population density, while the urban-industrial area is located in the industrial zone, and the suburban area is in the southern mountainous region. Due to the improved ecological environment and air quality, ecotourism has become popular in the suburbs. The selected observation points represent different locations and environments and may reflect different air quality levels in Ji'nan.

### Air pollution data

Air pollution data were collected from the Ji'nan Environmental Monitoring Center of Shandong Province, which monitored and analysed the air quality (data were obtained from Tenglong Jiang). Daily concentrations were measured for six air pollutants: particles with a diameter ≤ 2.5 µm (PM_2.5_) and particles with a diameter ≤ 10 µm (PM_10_), sulfur dioxide (SO_2_), carbon monoxide (CO), nitrogen dioxide (NO_2_), and ozone (O_3_).

### AQI calculation

The air quality index (AQI) is a dimensionless index that is calculated according to the Chinese ambient air quality standard (GB3095-2012), and the calculation includes the above six pollutants. The subindex of each pollutant is first calculated according to the fractional concentration and is labelled IAQI_p_^52^.

1 $${\mathrm{AQI}}_{\mathrm{i}} =\frac{{\mathrm{IN}}_{\mathrm{HI}}-{\mathrm{IN}}_{\mathrm{LO}}}{{\mathrm{B}}_{\mathrm{HI}}-{\mathrm{B}}_{\mathrm{LO}}}\times ({\mathrm{C}}_{\mathrm{i}}-{\mathrm{B}}_{\mathrm{LO}})+{\mathrm{IN}}_{\mathrm{LO}}$$

where C_i_ is the mass concentration of pollutant i, B_HI_ and B_LO_ are breakpoint concentrations greater and smaller than C_i_ in Table 1, and IN_HI_ and IN_LO_ are the air quality subindices corresponding to the B_HI_ and B_LO_ values. The overall AQI is the maximum AQIi, and the corresponding pollutant is the dominant pollutant. A high AQI indicates that serious and concentrated air pollution not only affects the outdoor activities of humans but also damages their health.

**Table 1:** Pollutant concentration limits.

AQI	PM_10_ (24-h average µg/m^3^)	PM_2.5_ (24-h average µg/m^3^)	SO_2_ (24-h average µg/m^3^)	NO_2_ (24-h average µg/m^3^)	CO (24-h average mg/m^3^)	O_3_ (8-h average µg/m^3^)
0	0	0	0	0	0	0
50	50	35	50	40	2	100
100	150	75	150	80	4	160
150	250	115	475	180	14	215
200	350	150	800	280	24	265
300	420	250	1600	565	36	800
400	500	350	2100	750	48	–
500	600	500	2620	940	60	–

### Observation period division

Our field observations were conducted from 15 January 2020 to 7 February 2020. Based on a local investigation in Ji'nan, we identified several important dates, such as 24 January (Chinese New Year’s Eve) and 24 January (the first day of the strictest lockdown in Shandong Province). The novel coronavirus was not confirmed as an epidemic in Shandong Province. Industrial operations and traffic continued to be normal in most regions in China during this period. In Shandong Province, we identified a pre-COVID period (15 January to 23 January 2020) and COVID lockdown period (24 January to 7 February 2020).

### Statistical analysis

In this paper, a comparative analysis was conducted to analyse the effects of the COVID-19 lockdown on the atmospheric environment at various monitoring stations. A preliminary analysis of the experimental data received from each monitoring station was performed to identify abnormal data and organize the data in a table based on 1-h averages. First, it is important to use time series to study the trends of the six pollutants. To compare the results obtained on different days, mean and standard deviation values were used. Second, in the COVID-19 lockdown period (including in the urban area), the statistical significance of the changes in the average daily concentrations of the six pollutants and the AQI was assessed. Contaminants were individually evaluated to identify possible autocorrelation in the sample data. The normality of the data was analysed, and transforms were performed as needed to satisfy the model assumptions. A bivariate Pearson correlation analysis of the pollutants and meteorological factors was also performed with a significance level of α = 0.05. Data analysis was conducted in SPSS 26.0 (SPSS Inc. IBM Corporation, Armonk, NY, USA). The map of Observation site (Fig. 1) was completed by the geostatistical analysis module of ArcGIS (version 10.3, https://developers.arcgis.com/). Plots were created in Origin 2020. Principal component analyses (PCAs) were performed separately for each of the periods and regions (two periods and three regions) by using Canoco 5.0.
